# Direct Medical Costs of 3 Reportable Travel-Related Infections in Ontario, Canada, 2012–2014

**DOI:** 10.3201/eid2508.190222

**Published:** 2019-08

**Authors:** Rachel D. Savage, Laura C. Rosella, Natasha S. Crowcroft, Maureen Horn, Kamran Khan, Laura Holder, Monali Varia

**Affiliations:** Women’s College Hospital, Toronto, Ontario, Canada (R.D. Savage);; University of Toronto, Toronto (R.D. Savage, L.C. Rosella, N.S. Crowcroft, K. Khan);; ICES, Toronto (R.D. Savage, L.C. Rosella, L. Holder);; Public Health Ontario, Toronto (N.S. Crowcroft);; Peel Public Health, Mississauga, Ontario, Canada (M. Horn, M. Varia);; St. Michael’s Hospital, Toronto (K. Khan)

**Keywords:** travel, hepatitis A, hepatitis A virus, malaria, *Plasmodium vivax*, *Plasmodium falciparum*, typhoid fever, *Salmonella enterica*, paratyphoid fever, costs and cost analysis, emigrants, immigrants, bacteria, viruses, parasites, Ontario, Canada, travel-related infections, VFR travelers

## Abstract

Immigrants traveling to their birth countries to visit friends or relatives are disproportionately affected by travel-related infections, in part because most preventive travel health services are not publicly funded. To help identify cost-effective policies to reduce this disparity, we measured the medical costs (in 2015 Canadian dollars) of 3 reportable travel-related infectious diseases (hepatitis A, malaria, and enteric fever) that accrued during a 3-year period (2012–2014) in an ethnoculturally diverse region of Canada (Peel, Ontario) by linking reportable disease surveillance and health administrative data. In total, 318 case-patients were included, each matched with 2 controls. Most spending accrued in inpatient settings. Direct healthcare spending totaled $2,058,196; the mean attributable cost per case was $6,098 (95% CI $5,328–$6,868) but varied by disease (range $4,558–$7,852). Costs were greatest for enteric fever. Policies that address financial barriers to preventive health services for high-risk groups should be evaluated.

Because of the rapid growth of air travel and immigration, more travelers worldwide are exposed to nonendemic infectious diseases (e.g., Zika, measles, malaria) than ever before (*1*–*3*). In Ontario, Canada, >3,000 travel-related infections are reported to public health annually (*4*); this number is an underestimate because not all sick persons seek healthcare treatment, especially while traveling, and not all conditions are diagnosed and reported. Immigrant travelers who return to their birth countries to visit friends or relatives are a substantial risk group (*5*). In Canada and elsewhere, regions with high proportions of immigrant travelers to South Asia and Africa have the highest rates of imported cases of hepatitis A, malaria, and enteric fever (*4*,*6*–*8*). The disproportionate burden of travel-related infections in immigrants has been attributed to their traveling to riskier destinations (*9*) and prolonged travel stays (*10*,*11*) but also to their poor uptake of pretravel health services (*10*,*12*–*14*).

Pretravel health consultations provide an opportunity to intervene and reduce travel-related infections (*14*). The Committee to Advise on Tropical Medicine and Travel recommends that nonimmune travelers going to developing countries receive the hepatitis A vaccine (*15*), travelers going to South Asia receive the typhoid vaccine (*16*), and travelers going to regions where malaria is endemic receive chemoprophylaxis (*17*) before traveling. Despite these recommendations, pretravel health services are generally not covered by provincial universal insurance plans, with few exceptions (*18*). Private health insurance can fill these gaps by providing partial or complete coverage for these services; however, many travelers, including those visiting friends or relatives (VFR), who are at greater risk for infection, often do not have private insurance. The cost of pretravel health services has been described by VFR travelers as a barrier (*9*,*19*–*22*). As a result, public health officials have advocated for universal coverage of pretravel health services to reduce the substantial public health resources required for the management of these imported cases (*6*,*23*).

The direct medical costs of reportable travel-related infections to healthcare systems has not been measured. The existing estimates were determined primarily by using inpatient settings or are considered outdated (*24*–*26*). As outbound travel and annual immigration targets continue to increase, evidence is needed to determine if policies are meeting the healthcare needs of an increasingly diverse population. Furthermore, mathematic and economic models require this information as inputs, so the lack of cost estimates has limited the development of these models. In this report, we sought to measure healthcare utilization and attributable medical costs of 3 key reportable travel-related infections in an ethnoculturally diverse region of Canada by linking public health reportable disease surveillance data with health administrative data.

## Materials and Methods

### Design and Setting

We received ethics approval (no. 31366) for this study from the University of Toronto Research Ethics Board (Toronto, Canada). We used a population-based, matched-cohort design to estimate attributable medical costs of incident cases of hepatitis A, malaria, and enteric fever from a healthcare payer perspective. This study was conducted in the Peel region of Ontario, one of Canada’s largest and most ethnoculturally diverse municipalities, which has ≈1.4 million residents, ≈50% of whom are foreign-born (*27*). South Asians are the largest visible minority in both the Peel region and Canada (*28*). For the purposes of our study, we needed to link reportable disease surveillance data with the health administrative data collected as part of Ontario’s government-funded, universal healthcare. Each data source independently would have been insufficient to achieve the study objective, and thus, the linkage represents a valuable feature of this study.

### Case-Patients

During the study period, reporting hepatitis A, malaria, and enteric fever (i.e., typhoid fever and paratyphoid fever) to public health authorities in Ontario was required by the Health Protection and Promotion Act (*29*). We identified the laboratory-confirmed hepatitis A, malaria, and enteric fever case-patients reported to Peel Public Health during January 1, 2012–December 31, 2014, using the integrated Public Health Information System (iPHIS). We excluded case-patients who were unable to be linked to the Ontario Registered Persons Database, which contains the demographic information of all persons issued an Ontario health card, and we compared linked and unlinked case-patients to identify potential sources of bias (*30*). Because reporting is known to be incomplete, we additionally identified Peel region residents with information in the health administrative datasets who were hospitalized with the following diagnostic codes from the International Classification of Diseases, 10th Revision, with Canadian Enhancements: hepatitis A (B15), malaria (B50–54), or typhoid and paratyphoid fever (A01). If hospitalizations were recurrent, we counted the series once (i.e., we considered all hospitalizations to be a part of the same case). We excluded case-patients identified in the health administrative datasets if their healthcare record indicated uncertainty of the diagnosis (*31*). Index dates were based on the iPHIS episode accurate date (i.e., the symptom onset date for 95% of case-patients and specimen collection date for the remainder) or the case-patient’s hospital admission date. Index dates for iPHIS case-patients were December 20, 2011–December 17, 2014. To account for delays between symptom onset and healthcare seeking, we included case-patients from hospitalization records if they were admitted during December 20, 2011–January 4, 2015. We chose to add this ≈3-week extension to the end of the accrual window because iPHIS case-patients had, on average, an 18-day delay between symptom onset and healthcare presentation.

### Controls

**Figure 1:**
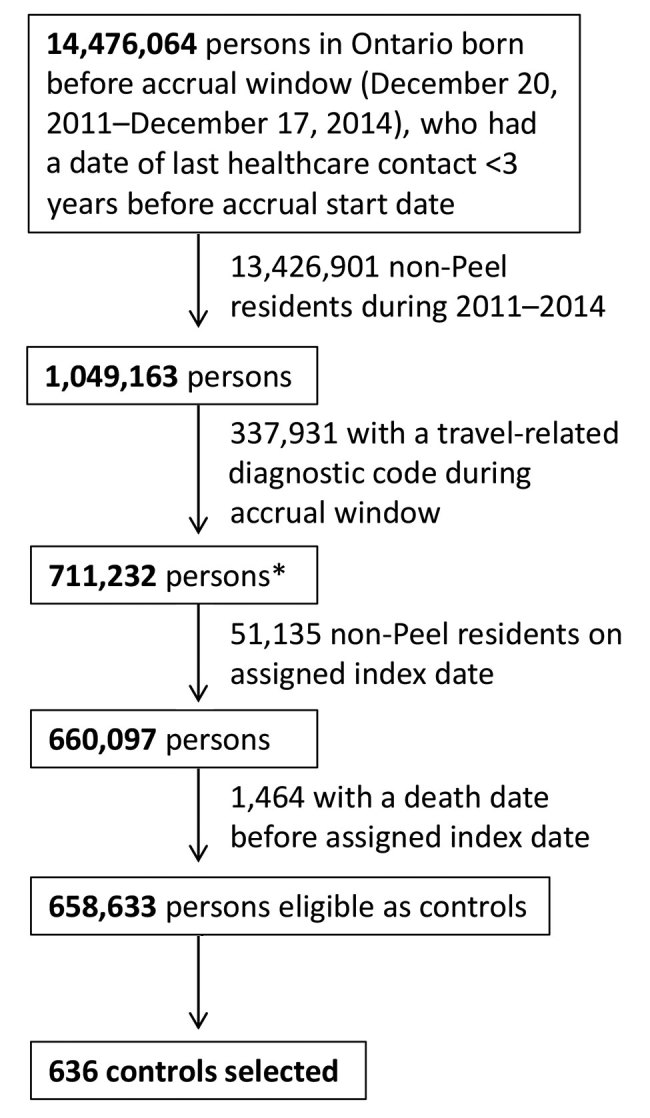
Flowchart of control selection in study of direct costs of hepatitis A, malaria, and enteric fever, Peel region, Ontario, Canada, 2011–2014. *Index date randomly assigned on the basis of the index date distribution of case-patients.

For each disease, we matched 2 controls per case-patient from a pool of eligible controls in the health administrative datasets. We used controls to determine the baseline medical costs so that we could calculate the costs attributable to travel. Controls were eligible if they were registrants of the Ontario Health Insurance Program, had contact with the healthcare system within the 3 years before their assigned index date, and resided in the Peel region (according to their postal code) during the period of study (Figure 1). We excluded persons from the pool of eligible controls if they had a travel-related diagnostic code during the study period (Table 1). We randomly assigned index dates to eligible controls according to the index date distribution of case-patients and then excluded controls who did not reside in the Peel region or had died as of their assigned index date.

**Table 1:** Diagnostic codes used to identify and exclude noneligible controls in study of direct costs of hepatitis A, malaria, and enteric fever, Peel region, Ontario, Canada, 2012–2014*

Disease	ICD-10-CA	OHIP
Hepatitis A	B15, B19, A09, A08.3–A08.5	070, 009, 079, 136, 787
Malaria	B50–B54, P37.3–P37.4, B64	062, 136, 781, 784, 787
Enteric fever	A01, A02.9, A02.1, A04.9, A05.9, A09, A49.9	002, 009, 003, 005, 136, 784, 787

*ICD-10-CA, International Classification of Diseases, 10th Revision, Canadian Enhancement; OHIP, Ontario Health Insurance Plan claims database.

### Matching and Covariates

For each disease, we matched controls to case-patients by index date (+60 days), age (+3 years), sex, neighborhood income quintile, foreign-born status, years in Canada if foreign-born (0–4, 5–9, or >10), and concurrent medical conditions. We estimated neighborhood income quintile by linking case-patients’ postal codes to existing average household income data for their neighborhoods and stratifying neighborhoods by quintile to generate neighborhood-specific income quintiles (*32*). We enhanced the study cohort by linking to the Ontario portion of the Immigration, Refugees, and Citizenship Canada Permanent Resident (IRCC-PR) database (1985–2012) (*33*). We defined case-patients as foreign-born if they had a country of birth outside of Canada recorded in iPHIS or if they had a record present in the IRCC-PR database. We designated controls as foreign-born solely using IRCC-PR data. To calculate years in Canada, we used the immigration date in the IRCC-PR database. For immigrants not yet captured in the IRCC-PR database (i.e., immigrants who landed in Ontario after 2012), we assigned their immigration date as the date 3 months before their first healthcare contact because new Ontario residents have a 3-month waiting period before they become eligible for the Ontario Health Insurance Program (*34*). For a small number of case-patients (n<6), we set their years in Canada to 0 because their index date preceded their first contact with healthcare. We determined the concurrent medical conditions of case-patients and controls during the 2 years before their index date by using the Johns Hopkins ACG System collapsed Aggregated Diagnostic Groups validated for use in Ontario (*35*,*36*). We further collapsed categories of like duration and severity of condition (i.e., unstable chronic conditions requiring medical [category 5] or specialty care [category 9], stable chronic conditions requiring medical [category 6] or specialty care [category 7]).

### Healthcare Utilization and Costs

We measured healthcare utilization and the medical costs that occurred up to 90 days after and including the index date; we chose this period on the basis of published estimates of illness duration (*37*). We probabilistically linked case-patients present in iPHIS to their health administrative data with unique identifiers and used a validated person-centered costing method developed for analyzing Ontario health administrative data to estimate direct medical costs (*38*). Those datasets were linked using unique encoded identifiers and analyzed at ICES (Toronto, Canada). The costing method used captured all relevant costs covered by the Ontario single-payer health insurance plan, including inpatient hospitalizations, emergency department (ED) visits, same-day surgery, dialysis, oncology clinic visits, fee-for-service physician and nonphysician services, non–fee-for-service physicians, prescription medications, laboratory services, rehabilitation, complex continuing care, long-term care, mental health inpatient stays, and home care services. We adjusted costs for inflation by using health sector–specific consumer price indices and reported all costs in 2015 Canadian dollars (Can).

### Analysis

We assessed the quality of matching by comparing case-patients and controls using the standardized difference (*d*); we used a *d* value of >0.10 to identify significant imbalances (*39*,*40*). We estimated attributable, 90-day per-person costs as the mean of the differences in costs among case-patient–control pairs (*41*). To account for the clustered nature of the data (i.e., 2 controls per case-patient), we used generalized estimating equations with an exchangeable correlation matrix to estimate 95% CIs (*41*). We stratified all attributable cost estimates by disease and setting and, for malaria, by infecting species. We categorized inpatient settings as inpatient hospitalizations, inpatient mental health hospitalizations, rehabilitation services, and complex continuing care; ED settings as ED visits and shadow billings to ED physicians; and the remainder of settings as outpatient settings. We performed analyses using SAS version 9.3 (https://www.sas.com).

### Sensitivity Analyses

To examine the effect of healthcare database selection and the time window on estimated costs, we repeated our analysis in 2 ways. In the first sensitivity analysis, we included only the cost calculations for expenditures considered most relevant to diagnosing and treating acute infections (i.e., inpatient hospitalizations, ED visits, same-day surgery, fee-for-service physician and nonphysician services, non–fee-for-service physicians, prescription medications, laboratory services). In the second, we limited the measurement of costs to a 60-day follow-up period.

## Results

During 2012–2014, a total of 289 cases of hepatitis A, malaria, or enteric fever were reported to Peel Public Health in case-patients linkable to the health administrative data, a linkage ratio of 90% (289/321). No deaths were identified among the case-patients. Unlinked case-patients (n = 32) were more likely than linked case-patients (n = 289) to be recent immigrants (i.e., immigrants who arrived in Ontario during the incubation period of their disease) or visitors (53.3% unlinked vs. 8.7% linked; p<0.001), but otherwise the linked and unlinked case-patients were similar in terms of sex, age, disease distribution, and foreign-born status. We identified an additional 29 case-patients in hospital records for a total case-patient cohort size of 318.

Compared with all (unmatched) eligible controls, case-patients were younger and a higher proportion were male (Table 2). Case-patients were also more likely than unmatched controls to be foreign-born, have acute or stable chronic medical conditions, and live in neighborhoods of lower income quintiles. Case-patients and matched controls were similar in terms of all matching variables, with the exception of missing neighborhood income quintile (Tables 2, 3).

**Table 2:** Characteristics of case-patients in study of direct costs of hepatitis A, malaria, and enteric fever and pools of unmatched and matched eligible controls, Peel region, Ontario, Canada, 2012–2014*

Variable	Case-patients, n = 318	Prematching			Postmatching	
		Controls, n = 658,633	*d*		Controls, n = 636	*d*
Age, y						
Mean ± SD	31 ± 21	37 ± 22	0.273		31 ± 21	0.001
Median (IQR)	30 (12–47)	36 (20–53)	0.268		30 (12–47)	0.001
<1	0	9,694 (1.5)	0.173		0	0
1–4	21 (6.6)	27,381 (4.2)	0.109		42 (6.6)	0
5–9	36 (11.3)	40,692 (6.2)	0.183		75 (11.8)	0.015
10–14	33 (10.4)	41,235 (6.3)	0.149		63 (9.9)	0.016
15–24	43 (13.5)	93,870 (14.3)	0.021		85 (13.4)	0.005
25–49	120 (37.7)	245,090 (37.2)	0.011		240 (37.7)	0
50–74	57 (17.9)	167,999 (25.5)	0.185		115 (18.1)	0.004
>75	8 (2.5)	32,672 (5.0)	0.129		16 (2.5)	0
Sex						
F	123 (38.7)	321,510 (48.8)	0.205		252 (39.6)	0.019
M	195 (61.3)	337,123 (51.2)	0.205		384 (60.4)	0.019
Neighborhood income quintile						
1, lowest	70 (22.0)	103,751 (15.8)	0.160		133 (20.9)	0.027
2	88 (27.7)	109,728 (16.7)	0.268		169 (26.6)	0.025
3	94 (29.6)	121,601 (18.5)	0.262		203 (31.9)	0.051
4 or 5, highest	60–70 (S)	159,348 (24.2)	0.231		131 (20.6)	0.027
Missing	<6 (S)	356 (0.1)	0.150		0	0.160
Foreign-born, database						
IRCC-PR†	183 (57.5)	73,564 (11.2)	1.119		449 (70.6)	0.275
iPHIS or IRCC-PR‡	227 (71.4)	73,564 (11.2)	1.546		449 (70.6)	0.017
Years in Canada, no./total (%)						
0–4	58/227 (25.6)	13,535/73,564 (18.4)	0.170		113/449 (25.2)	0.009
5–9	56/227 (24.7)	15,868/73,564 (21.6)	0.070		114/449 (25.4)	0.017
>10	113/227 (49.8)	44,161/73,564 (60.0)	0.210		222/449 (49.4)	0.007
Concurrent medical condition§						
1	240 (75.5)	351,379 (53.3)	0.475		475 (74.7)	0.018
2	210 (66.0)	313,777 (47.6)	0.378		420 (66.0)	0
3	178 (56.0)	286,375 (43.5)	0.252		359 (56.4)	0.010
4	28 (8.8)	33,259 (5.0)	0.148		59 (9.3)	0.016
5 and 9	44 (13.8)	90,116 (13.7)	0.004		118 (18.6)	0.008
6 and 7	107 (33.6)	164,535 (25.0)	0.191		222 (34.9)	0.007
8	12 (3.8)	39,357 (6.0)	0.102		23 (3.6)	0.008
10	63 (19.8)	135,170 (20.5)	0.018		127 (20.0)	0.004
11	119 (37.4)	216,829 (32.9)	0.094		216 (34.0)	0.072
12	12 (3.8)	15,640 (2.4)	0.081		24 (3.8)	0

*Values are no. (%) except as indicated. *d*, standardized difference; iPHIS, integrated Public Health Information System; IQR, interquartile range; IRCC-PR, Immigration, Refugee and Citizenship Canada Permanent Resident; S, suppressed per ICES reidentification risk assessment policy. †Case-patients and controls were designated as foreign-born if they had a record in the IRCC-PR database. ‡Case-patients were designated as foreign-born if country of birth was listed as outside of Canada in iPHIS, or they had a record in the IRCC-PR database; controls were designated as foreign-born if they had a record in the IRCC-PR database. §Listed by John Hopkins collapsed Aggregated Diagnostic Group: 1 (acute minor), 2 (acute major), 3 (likely to recur), 4 (asthma), 5 and 9 (chronic unstable), 6 and 7 (chronic stable), 8 (eye and dental), 10 (psychosocial), 11 (preventive and administrative), and 12 (pregnancy)..

**Table 3:** Characteristics of case-patients and matched controls in study of direct costs of hepatitis A, malaria, and enteric fever, by disease, Peel region, Ontario, Canada, 2012–2014*

Variable	Hepatitis A				Malaria				Enteric fever		
	Case-patients, n = 55	Controls, n = 110	*d*		Case-patients, n = 122	Controls, n = 244	*d*		Case-patients, n = 141	Controls, n = 282	*d*
Age, y											
Median ± IQR	24 ± 21	24 ± 21	0		40 ± 19	40 ± 19	0.001		26 ± 19	26 ± 19	0.002
Mean (SD)	19 (10–32)	19 (10–32)	0.003		43 (27–55)	43 (27–55)	0.002		25 (10–40)	25 (9–40)	0.001
0–9	13 (23.6)	23 (23.6)	0		10 (8.2)	20 (8.2)	0		34 (24.1)	71 (25.1)	0.025
10–14	7 (12.7)	14 (12.7)	0		7 (5.7)	14 (5.7)	0		19 (13.5)	35 (12.4)	0.032
15–24	17 (30.9)	34 (30.9)	0		10 (8.2)	20 (8.2)	0		16 (11.3)	31 (11.0)	0.011
25–49	12 (21.8)	24 (21.8)	0		54 (44.3)	107 (43.9)	0.008		54 (38.3)	109 (38.7)	0.007
>50	6 (11.0)	12 (11.0)	0		41 (33.6)	83 (34.0)	0.008		18 (12.8)	36 (12.8)	0
Sex											
F	27 (49.1)	54 (49.1)	0		37 (30.3)	78 (32.0)	0.035		59 (41.8)	120 (42.6)	0.014
M	28 (50.9)	56 (50.9)	0		85 (69.7)	166 (68.0)	0.035		82 (58.2)	162 (57.4)	0.014
Income quintile											
1, lowest	9 (16.4)	15 (13.6)	0.076		36 (29.5)	68 (27.9)	0.036		25 (17.7)	50 (17.7)	0
2	6 (10.9)	10 (9.1)	0.061		39 (32.0)	77 (31.6)	0.009		43 (30.5)	82 (29.1)	0.031
3	17 (30.9)	36 (32.7)	0.039		20–25 (S)	56 (23.0)	0.060		52 (36.9)	111 (39.4)	0.051
4 and 5, highest	23 (41.8)	49 (44.5)	0.055		20–25 (S)	43 (17.6)	0.033		15–20 (S)	39 (13.8)	0.010
Missing	0	0	0		<6 (S)	0	0.183		<6 (S)	0	0.170
Foreign-born	30 (54.5)	58 (52.7)	0.036		101 (82.8)	200 (82.0)	0.022		96 (68.1)	191 (67.7)	0.008
Years in Canada, no./total (%)											
0–4	6/30 (20.0)	10/58 (17.2)	0.071		29/101 (28.7)	63/200 (31.5)	0.061		23/96 (24.0)	40/191 (20.9)	0.072
5–9	10/30 (33.3)	20/58 (34.5)	0.024		21/101 (20.8)	40/200 (20.0)	0.020		25/96 (26.0)	54/191 (28.3)	0.050
>10	14/30 (46.7)	28/58 (48.3)	0.032		51/101 (50.5)	97/200 (48.5)	0.040		48/96 (50.0)	97/191 (50.8)	0.016
Concurrent medical conditions†											
1	43 (78.2)	84 (76.4)	0.043		84 (68.9)	166 (68.0)	0.018		113 (80.1)	225 (79.8)	0.009
2	37 (67.3)	75 (68.2)	0.019		78 (63.9)	161 (66.0)	0.043		95 (67.4)	184 (65.2)	0.045
3	32 (58.2)	68 (61.8)	0.074		59 (48.4)	118 (48.4)	0		87 (61.7)	173 (61.3)	0.007
4	<6 (S)	6 (5.5)	0		7 (5.7)	17 (7.0)	0.050		18 (12.8)	36 (12.8)	0
5 and 9	11 (20.0)	19 (17.3)	0.070		25 (20.5)	56 (23.0)	0.060		24 (17.0)	43 (15.2)	0.048
6 and 7	13 (23.6)	25 (22.7)	0.022		49 (40.2)	98 (40.2)	0		50 (35.5)	99 (35.1)	0.007
8	<6 (S)	<6 (S)	0		<6 (S)	9 (3.7)	0.022		7 (5.0)	12 (4.3)	0.034
10	12 (21.8)	26 (23.6)	0.043		24 (19.7)	44 (18.0)	0.042		27 (19.1)	57 (20.2)	0.027
11	23 (41.8)	42 (38.2)	0.074		37 (30.3)	69 (28.3)	0.045		59 (41.8)	105 (37.2)	0.094
12	<6 (S)	<6 (S)	0		<6 (S)	12 (4.9)	0.040		<6 (S)	8 (2.8)	0.040

*Values are no. (%) except as indicated. *d*, standardized difference; IQR, interquartile range; S, suppressed per ICES reidentification risk assessment policy. †Listed by John Hopkins collapsed Aggregated Diagnostic Group: 1 (acute minor), 2 (acute major), 3 (likely to recur), 4 (asthma), 5 and 9 (chronic unstable), 6 and 7 (chronic stable), 8 (eye and dental), 10 (psychosocial), 11 (preventive and administrative), and 12 (pregnancy).

Most (>90%) case-patients had travel-associated illnesses (Table 4); of these case-patients, most (50%) traveled to India, and more than half (63%) reported traveling to visit friends or relatives. A similar proportion of children <16 years of age (57% [43/76]) also reported traveling to visit friends or relatives, although this proportion varied by disease (47% [8/17] for hepatitis A, 40% [6/15] for malaria, 66% [29/44] for enteric fever). Most of the case-patients identified as recent immigrants received malaria diagnoses, and of these, 65% (13/20) were caused by *Plasmodium vivax*, probably representing relapsed malaria rather than primary disease acquired by travel. Case-patients with *P. falciparum* malaria primarily traveled to West Africa (83%, 40/48), and all case-patients with *P. vivax* malaria had traveled to India (58%, 15/26) or Pakistan (42%, 11/26). Few case-patients reported having a pretravel health consultation.

**Table 4:** Travel-related characteristics of case-patients with hepatitis A, malaria, or enteric fever reported to public health, Peel region, Ontario, Canada, 2012–2014*

Characteristic	No. (%) case-patients			
	Hepatitis A	Malaria	Enteric fever	Overall
Travel associated†				
Yes	39 (79.6)	103 (91.2)	120 (94.5)	262 (90.7)
No or unknown	10 (20.4)	10 (8.8)	7 (5.5)	27 (9.3)
Region of birth†				
South Asia	10 (20.4)	38 (33.6)	78 (61.4)	126 (43.6)
North America	14 (28.6)	6 (5.3)	35 (27.6)	55 (19.0)
West Africa	0	29 (25.7)	0	29 (10.0)
Other or missing	25 (51.0)	40 (35.4)	14 (11.0)	79 (27.3)
Primary travel country‡				
India	11 (29.0)	16 (19.5)	92 (78.6)	119 (50.2)
Pakistan	14 (36.8)	11 (13.4)	19 (16.2)	44 (18.6)
Nigeria	0	20 (24.4)	0	20 (8.4)
Ghana	0	19 (23.2)	0	19 (8.0)
Other or missing	13 (34.2)	16 (19.5)	6 (5.1)	35 (14.8)
Purpose of travel‡§				
Visiting friends or relatives	18 (47.4)	45 (54.9)	87 (74.4)	150 (63.3)
Leisure, business, or other	<6 (S)	13 (15.9)	11 (9.4)	25–29 (S)
Missing	17 (44.7)	27 (32.9)	24 (20.5)	68 (28.7)
Pretravel health consultation‡				
Yes	<6 (S)	14 (17.1)	10 (8.5)	25–29 (S)
No or unknown	33–38 (S)	68 (82.9)	107 (91.5)	208–213 (S)

*S, suppressed per ICES reidentification risk assessment policy. †Includes recent immigrants (i.e., immigrants who arrived in Ontario during the incubation period of their disease) and visitors: hepatitis A (n = 49), malaria (n = 113), enteric fever (n = 127), and overall (n = 289). ‡Does not include recent immigrants and visitors: hepatitis A (n = 38), malaria (n = 82), enteric fever (n = 117), and overall (n = 237). §Not mutually exclusive.

Of the 318 case-patients, 197 (61.9%) were hospitalized, 232 (73.0%) visited the ED, and 298 (93.7%) visited a physician (i.e., family or general practice physician or specialist) for their illness. However, a total of 225 hospitalizations, 429 ED visits, and 4,831 unique physician visits occurred (Table 5); more than half of these encounters were for enteric fever diagnoses or treatment.

**Table 5:** Healthcare utilization by type of healthcare visit among case-patients with travel-related hepatitis A, malaria, or enteric fever, stratified by foreign-born status, Peel region, Ontario, Canada, 2012–2014

Population	No. hospitalizations	Length of hospital stay, d			No. emergency department visits	No. physician visits	No. outpatients
		Mean	Median	Range			
All, n = 318	225	4	3	0–42	429	4,831	1,317
Hepatitis A	31	4	3	0–14	61	742	208
Malaria	75	3	2	0–42	118	1,488	427
Enteric fever	119	5	4	0–34	250	2,601	682
Canada-born, n = 91							
All	71	4	3	0–14	126	1,318	369
Hepatitis A	15	3	2	0–6	27	276	78
Malaria	16	2	2	0–8	30	293	85
Enteric fever	40	6	5	1–14	69	749	206
Foreign-born, n = 227							
All	154	4	3	0–42	303	3,513	948
Hepatitis A	16	5	4	0–14	34	466	130
Malaria	59	3	2	0–42	88	1,195	342
Enteric fever	79	5	4	0–34	181	1,852	476

The overall cost of the 318 travel-related infections that occurred during the 3-year study period in Peel was $2,058,196 (Table 6). Extrapolating the estimated mean cost of these infections per case-patient for the Peel region to the Ontario case counts (*42*) amounted to a total of $7,870,341 in direct healthcare spending. More than one quarter (26.2%) of the healthcare costs of hepatitis A, malaria, and enteric fever in Ontario were accrued in the Peel region, despite this region comprising only 10.3% of the Ontario population (*28*).

**Table 6:** Total direct and attributable medical costs of hepatitis A, malaria, and enteric fever, Peel region, Ontario, Canada, 2012–2014*

Category	Peel region						Ontario	
	No. case-patients	Total direct costs, $	Cost per case-patient, $, mean (range)	Cost per control, $, mean (range)	Attributable cost (95% CI), $		No. case-patients	Total direct costs, $†
Overall 90-d cost	318	2,058,196	6,472 (0–59,358)	375 (0–42,213)	6,098 (5,328–6,868)		1,216	7,870,341
By disease								
Hepatitis A	55	306,707	5,576 (0–59,358)	560 (0–42,213)	5,016 (2,414–7,619)		300	1,672,944
Malaria	122	613,488	5,029 (0–38,556)	471 (0–26,598)	4,558 (3,557–5,558)		612	3,077,497
* Plasmodium vivax*	43	230,487	ND	ND	4,812 (2,675–6,949)		ND	ND
*P*. *falciparum*	59	297,106	ND	ND	4,743 (3,588–5,898)		ND	ND
Other	20	85,895	ND	ND	3,463 (1,397–5,528)		ND	ND
Enteric fever	141	1,138,002	8,071 (0–33,563)	219 (0–13,824)	7,852 (6,812–8,893)		304	2,453,566
Restricted to most relevant costs								
Hepatitis A	55	233,771	4,250 (0–23,048)	266 (0–10,163)	3,984 (2,823–5,145)		300	1,275,117
Malaria	122	611,030	5,008 (0–37,335)	361 (0–17,626)	4,647 (3,593–5,701)		612	3,065,171
Enteric fever	141	1,029,311	7,300 (0–33,563)	211 (0–13,824)	7,089 (6,097–8,081)		304	2,219,224

*Cost given in 2015 Canadian dollars. Attributable cost indicates the additional cost for each case-patient. ND, not determined. †Extrapolated by multiplying the number of cases in the province reported to Public Health Ontario for the same period by the mean cost per case-patient estimated by using Peel region data.

**Figure 2:**
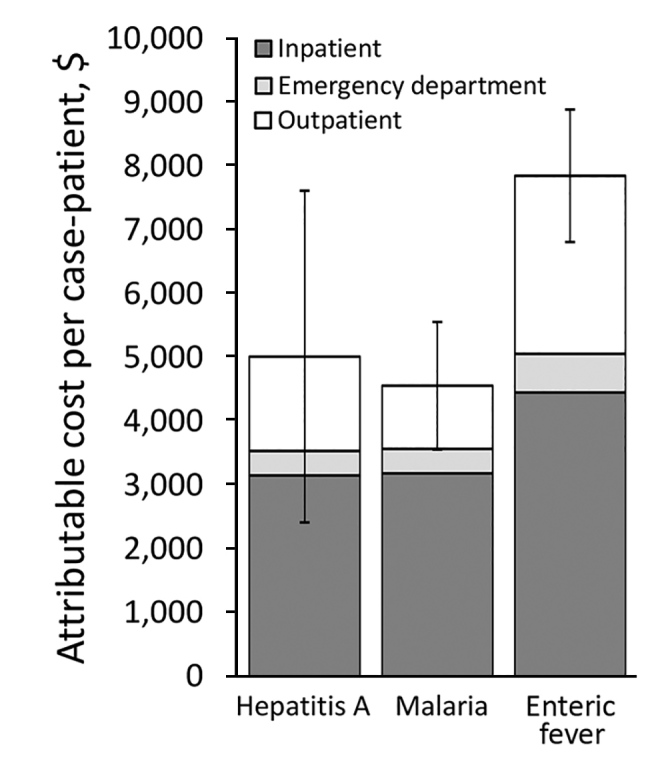
Attributable 90-day healthcare costs in study of direct costs of hepatitis A, malaria, and enteric fever, by disease and healthcare sector, Peel region, Ontario, Canada, 2012–2014. Cost is given in 2015 Canadian dollars. Error bars indicate 95% CIs.

The average healthcare spending per case-patient ($6,472) was >17 times the cost per control ($375), for an attributable additional cost of $6,098 (95% CI $5,328–$6,868) per case-patient (Table 6). Costs varied by disease and were greatest for enteric fever. Attributable healthcare costs were primarily accrued in inpatient settings, which accounted for 57%–70% of costs across infections (Figure 2), followed by outpatient (22%–36%) and ED settings (8%).

Attributable costs were robust across sensitivity analyses. When restricted to the most relevant healthcare costs, costs were ≈$1,000 less for hepatitis A but remained similar for the other infectious diseases (Table 6). A shorter follow-up period of 60 days similarly did not substantially change cost estimates ($5,859, 95% CI $5,126–$6,592). To examine how costs were affected by our decision to include the small number of case-patients with locally acquired hepatitis A and those for which travel exposure was unknown (n = 10), we conducted a post hoc sensitivity analysis excluding these case-patients. The overall attributable cost remained relatively unchanged at $5,942 (95% CI $5,247–$6,637) per case-patient, and the mean hepatitis A–specific attributable cost was reduced to $3,710 (95% CI 95% CI $2,044–$5,376) per case-patient.

## Discussion

By linking reportable disease surveillance data with health administrative data, this study provides comprehensive, population-based estimates of the medical costs of 3 reportable travel-related infections. Total medical costs were >$2 million in the Peel region of Ontario during our 3-year study period; scaled up to the provincial level, we estimated close to $8 million in healthcare spending for the 1,216 reported cases in Ontario that occurred over the same period. Attributable 90-day medical costs ranged from $4,558 for malaria to $7,852 for enteric fever; these estimates are in line with total per capita health expenditures in Ontario of $6,584 in 2018 (*43*), highlighting the substantial resources required for diagnosis and treatment of these infections. Costs were primarily driven by care provided in inpatient settings and represent mostly avoidable healthcare spending, considering that safe and effective medical interventions (e.g., pretravel health consultations, immunization, chemoprophylaxis) are available to prevent infection and clinical disease.

To the best of our knowledge, the comprehensive medical costs of hepatitis A, malaria, and enteric fever have not been estimated elsewhere. In London, England, where rates of reportable travel-related infections are high and reflective of an ethnically diverse population, inpatient costs have been estimated at £1,375 (≈2015 Can $2,300) per admission for malaria and £1,976 (≈2015 Can $3,300) per admission for typhoid (*25*,*26*). These values are comparable, albeit slightly lower than the costs reported in our study, probably because of their restriction to the inpatient setting and lower healthcare costs in the United Kingdom (*38*,*44*). In both settings, costs of enteric fever were greater than those of malaria. This difference might be attributable to the poor sensitivity of available microbiological tests for the detection of *Salmonella enterica* serovars Typhi and Paratyphi, which can lead to repeated testing and delays in receiving appropriate treatment (*45*). Emerging antimicrobial resistance also contributes to costs. In a retrospective chart review of a large, tertiary care pediatric center in Toronto, only 40% of isolates were found to be fully susceptible to the drugs typically used to treat enteric fever, and 64% of patients needed to be recalled to the hospital after positive blood cultures (*46*). Improving diagnostics for enteric fever is critical to reducing hospitalizations and associated healthcare spending.

Although public health costs related to case and contact management are substantial, they are often excluded because they are not routinely or systematically tracked. Omission of these expenditures can lead to underestimates of disease burden, particularly for infections with hepatitis A virus and *S. enterica* serovars Typhi and Paratyphi, which require follow-up and resource-intensive postexposure prophylaxis (in cases of hepatitis A) to prevent secondary transmission (*47*). In 2014, Peel Public Health tracked staff hours spent to manage reported travel-related cases and estimated personnel costs of Can $3,500 per case for hepatitis A, Can $3,300 per case for enteric fever, and Can $40 per case for malaria (M. Varia, unpub. data). These estimates are in line with reports from the United States (US $3,221 per hepatitis A case) (*48*). However, costs can be >US $40,000 when including the cost of immunoglobulin and vaccines (*49*).

Overall, >70% of the case-patients in our study were foreign-born, suggesting that more needs to be done to ensure equitable access to these interventions for those who are at greatest risk. Although public funding of pretravel health services for VFR travelers might eliminate cost-related barriers, other factors need to be considered and addressed to effectively reduce disease burden in this population. Qualitative studies have found that social contexts and relationships are mediators of the choices and behaviors of VFR travelers (*20*,*22*). Unlike business or leisure travelers, VFR travelers often stay with family members when visiting and are part of collectivist cultures that place a strong emphasis on acting in the best interest of the family rather than an individual member. These cultural value systems might influence VFR travelers to make decisions to maintain family harmony at the expense of personal health and to go against medical or public health advice to be able to fully participate in cultural activities or rituals that are of value to them (*20*). They also might need to travel in rushed circumstances to attend to a sick or dying relative. To be effective, public health interventions need to consider the complex social environment in which VFR travelers make decisions. Interventions could include leveraging family in peer-education programs, providing culturally appropriate strategies for making safe food and drink choices that are respectful of hosts and enable travelers to participate in valued rituals, and developing streamlined planning resources for rushed travelers.

Because we took the perspective of the healthcare payer, we were unable to estimate costs incurred by new immigrants not yet eligible for publicly funded healthcare. The financial burden, emotional hardship, and negative health impact of the 3-month waiting period policy have been highlighted in a qualitative study (*50*). Whether and how new immigrants access care for reportable travel-related infections and subsequent risks for secondary transmission merits further investigation.

Total costs could have also been underestimated if relevant healthcare encounters of case-patients preceded the index date. The costing method we used, though comprehensive, did not include medical costs incurred by community health centers. Because these centers provide care to a small proportion (1%) of Ontario’s population, their exclusion was not anticipated to appreciably change our results. Our analysis also does not consider costs related to secondary cases or outbreaks, although no locally identifiable outbreaks of hepatitis A or enteric fever occurred in the Peel region during the study period.

For pragmatic reasons, our analysis focused on 3 reportable travel-related infections. The true burden of preventable travel-related infections on the healthcare system is expected to be much larger. Likewise, this analysis does not include healthcare costs for other adverse health events associated with travel, such as events related to concurrent medical conditions, incubating disease events, trauma, and heat- and smog-related illnesses that likely also substantially affect travelers. Although case-patients and controls were comparable by key confounding variables available in the health administrative datasets, they might have differed by other factors that were not measurable with the existing data (e.g., factors that influence healthcare-seeking behaviors and costs), which could have led to an overestimation or underestimation of the attributable cost. Last, we estimated the costs of reportable travel-related infections in a VFR traveler population that was primarily foreign-born and traveling to India and Pakistan. Findings might not be generalizable to other settings with different traveler types and travel patterns.

In conclusion, we found that the attributable medical costs of 3 key reportable travel-related infections were substantial and concentrated in the Peel region of Ontario among immigrants who traveled to visit friends or relatives. Our results could be used to parameterize economic evaluations aimed at determining whether subsidizing or eliminating costs of pretravel health services for high-risk travelers or other policies might be cost-effective. As more and more citizens travel and have links to developing countries through birth or parentage, policymakers must consider equitable strategies that are responsive to the evolving health needs of their populations.
